# Development and psychometric properties of a five-language multiperspective instrument to assess clinical decision making style in the treatment of people with severe mental illness (CDMS)

**DOI:** 10.1186/1471-244X-13-48

**Published:** 2013-02-04

**Authors:** Bernd Puschner, Petra Neumann, Harriet Jordan, Mike Slade, Andrea Fiorillo, Domenico Giacco, Anikó Égerházi, Tibor Ivánka, Malene Krogsgaard Bording, Helle Østermark Sørensen, Arlette Bär, Wolfram Kawohl, Sabine Loos

**Affiliations:** 1 Department of Psychiatry II, Ulm University, Ludwig-Heilmeyer-Str. 2, Günzburg, 89312, Germany; 2 King’s College London, Section for Recovery, Institute of Psychiatry, London, U.K; 3 Department of Psychiatry, Second University of Naples, Naples, Italy; 4 Department of Psychiatry, University of Debrecen Medical and Health Science Center, Debrecen, Hungary; 5 Unit for Psychiatric Research, Aalborg Psychiatric Hospital, Aalborg University Hospital, Aalborg, Denmark; 6 Department of General and Social Psychiatry, University of Zurich, Zurich, Switzerland

## Abstract

**Background:**

The aim of this study was to develop and evaluate psychometric properties of the Clinical Decision Making Style (CDMS) scale which measures general preferences for decision making as well as preferences regarding the provision of information to the patient from the perspectives of people with severe mental illness and staff.

**Methods:**

A participatory approach was chosen for instrument development which followed 10 sequential steps proposed in a current guideline of good practice for the translation and cultural adaptation of measures. Following item analysis, reliability, validity, and long-term stability of the CDMS were examined using Spearman correlations in a sample of 588 people with severe mental illness and 213 mental health professionals in 6 European countries (Germany, UK, Italy, Denmark, Hungary, and Switzerland).

**Results:**

In both patient and staff versions, the two CDMS subscales “Participation in Decision Making” and “Information” reliably measure distinct characteristics of decision making. Validity could be demonstrated to some extent, but needs further investigation.

**Conclusions:**

Together with two other five-language patient- and staff-rated measures developed in the CEDAR study (ISRCTN75841675) – “Clinical Decision Making in Routine Care” and “Clinical Decision Making Involvement and Satisfaction” – the CDMS allows empirical investigation of the complex relation between clinical decision making and outcome in the treatment of people with severe mental illness across Europe.

## Background

Decision-making in health care has been conceptualized as a process taking place between patient and health professional on a continuum between “paternalistic”, “shared”, and “informed” [1,2]. Shared decision making has received much attention in research and practice since its first mention 30 years ago [3]. Substantial evidence has accumulated in recent decades for clinical decision making in acute (e.g. heart attack, stroke) and long-term physical conditions (e.g. cancer and fibromyalgia) [4-10]. Shared decision making has been termed an ethical imperative [11] and is recommended in guidelines for the treatment of people with schizophrenia [12].

However, knowledge about clinical decision making in the treatment of people with mental illness is still limited. Most importantly, apart from a few studies [13], little is known about the relation between clinical decision making processes and outcome in this population. Knowledge gaps relating to investigating this link have been identified, including: (a) descriptive research and instrument development focussing on how decisions are actually made in routine care; (b) the development of measures to characterize decision making processes, especially in people with long-term mental disorder; and (c) the measurement of both patients’ and professionals’ decision making styles and how these are enacted in decision making encounters [14-16].

Published scales measuring decision making have been summarised in four recent reviews; three with a focus on shared decision making [16-19] and one specifically examining professionals’ perceptions of decision making [20]. Scales identified in these reviews measured a wide range of aspects of clinical decision making, which were thematically grouped by the authors of these reviews: “decision making needs”, “decision support”, and “evaluation of process and outcome” by Simon and colleagues [17]; “values and preferences”, “information and communication”, and “other” by Dy [18]; and “antecedents”, “process” and “outcomes” by Scholl and colleagues [19].

However, psychometric properties of most measures, especially validity, have not yet been sufficiently demonstrated [17,19]. Furthermore, many instruments assess preferences at a high level of abstraction, while little is known about actual decision behaviour in routine care [18]. Moreover, by exclusively assessing decision making from the perspective of the patient, instrument development has largely ignored reciprocity as a defining feature of decision making [1,21-23]. For example, even though some instruments to assess decision making from the perspective of health professionals have been developed [20], there is a lack of parallel versions with an explicit focus on the reciprocal assessment of decision making [17,19] from both patient and professional perspectives.

With a few exceptions (e.g. [24]), instruments used to assess decision making in the treatment of people with mental illness have been developed in samples of people with physical conditions (mainly cancer). However, there is emerging evidence that psychometric properties of decision making measures substantially vary by illness and treatment variables [25]. Thus, untested use of an instrument in populations other than the original target sample may be problematic. Nevertheless, some scales developed for other conditions have been successfully validated for assessing decision making in the treatment of people with mental illness, e.g. [26,27]. Notably the “Autonomy Preference Index” (API) [28] consisting of two subscales (15 item “decision-making preferences” and 8-item “information-seeking preferences”) has been widely used in mental health research [29-31]. Internal consistency (Cronbach’s α) of the original version [28] examined in 312 general medicine patients was .82 for both subscales, and test-retest reliability after two weeks in a subsample (N = 50) was .84 for the decision making preferences subscale, and .83 for the information seeking preferences subscale. For an abridged version of the API decision-making preferences subscale (6 items), a German study reported internal consistencies (Cronbach’s α) of .79 for GP patients with depression(N = 230), and of .59 for psychiatric inpatients with schizophrenia (N = 120) [32]. In a confirmatory factor analysis, three items showed questionable reliability in a sample of 1,592 patients with various conditions including 186 people with depression [33].

In order to measure key aspects of clinical decision making in the routine care for people with severe mental illness, the *CEDAR* study developed patient and staff versions of three new instruments, to measure: clinical decision making style; key elements of clinical decision making in routine care; and clinical decision making involvement and satisfaction.

This paper reports on the development and translation of the *Clinical Decision Making Style Scale* (CDMS), and investigates its psychometric properties (internal consistency, validity, and test-retest reliability) in a sample of 588 people with severe mental illness and 213 mental health professionals from six European countries. The development and psychometric properties of the *Clinical Decision Making Involvement and Satisfaction Scale* (CDIS) and the *Clinical Decision Making in Routine Care Scale* (CDRC) are reported elsewhere [34,35].

## Methods

### Instrument development

Development of the *Clinical Decision Making Style Scale* (CDMS) followed the International Society for Pharmacoeconomics and Outcomes Research (ISPOR) Task Force principles of good practice for the translation and cultural adaptation of patient-reported outcome measures [36]. The ISPOR Framework identifies ten sequential steps: 1 preparation; 2 forward translation; 3 reconciliation; 4 back translation; 5 back translation review; 6 harmonisation; 7 cognitive debriefing; 8 review of cognitive debriefing results and finalisation; 9 proof-reading; and 10 final report. We refer to these steps as ISPOR 1 to ISPOR 10 respectively.

Focus groups were held in ISPOR 1 and 7. Focus groups are widely used to examine people’s experience with illness and health services. They especially aim at enabling vulnerable people to freely express their views in the format of a moderated group discussion [37-39]. Patient participants of focus groups were convenience samples of native speaker adults aged 18-60 using local non-forensic mental health services. Staff participants were workers in these services. Focus groups were held in non-clinical settings, and moderated and co-facilitated by two CEDAR research workers who ensured that all responders had sufficient opportunity to air their views and that non-verbal group dynamics were noted. All focus groups were audiotaped and fully transcribed.

#### Development of the source language CDMS (ISPOR 1)

At the first CEDAR study meeting in May 2009, Ulm research workers presented results of an extensive literature search on the instruments with a special focus on identifying scales to be considered candidates for inclusion. Presentations were discussed by the study group including advisory board member, which informed the drafting of a topic guide for the first round of focus groups. Subsequently, six focus groups (4 with 23 patients altogether, 2 with 8 clinicians altogether) were held by researchers in Ulm to explore the conceptual understanding of clinical decision-making. Topics covered included the experience of making decisions as well as level of involvement and satisfaction with the process during the last treatment session. Procedures and results of these focus groups have been reported in detail elsewhere [40]. Subsequently the patient-rated “Autonomy Preference Index” (API) [28] was chosen as the basis of the CDMS, from which the Ulm study team produced a parallel staff version. Both versions were in English. Permission to use the instrument was granted by the author of the API (J. Ende).

#### Development of the target language CDMS (ISPOR 2-10)

The API was forward translated from English into the four other study languages (German, Italian, Hungarian, and Danish; Switzerland used the German version) (ISPOR 2). Forward translations in each centre were done by native speakers of the target language who were familiar with the concepts of the instrument. Assistance from professional translators was drawn upon as needed, and where more than one forward translations was produced independently of one another at a study centre, these were compared and merged into one single forward translation (ISPOR 3). Subsequently, in each study centre one person fluent in the source language who had not been involved into the forward translation(s) carried out a blind (without seeing the source) back translation into the source language (ISPOR 4). Back translations were then compared to the original by CEDAR team members who were English native speakers (ISPOR 5), and potential discrepancies were discussed with the key in-country person and corrected as needed (ISPOR 6). Patient and staff versions of instrument drafts were then subjected to a total of 17 focus groups (9 with 33 patients altogether, and 8 with 31 key workers altogether) at all study sites in order to test alternative wording and to check understandability, interpretation, feasibility, and cultural relevance of the translations (ISPOR 7). Review of cognitive debriefing results based upon reports of previous step to the Ulm study centre resulted in some final amendments including changes in text and omission of some items in order to arrive at short and understandable measures. These changes were: (i) omission of three items relating to information-seeking (“You should understand completely what is happening inside your body as a result of your illness”; “Even if the news is bad, you should be well informed”; “Information about your illness is as important to you as treatment”); (ii) some changes in wording (e.g. “clinician” instead of “doctor”, “I” instead of “you”); (iii) replacement of the content of the three clinical vignettes which in the original API relate to physical conditions (upper respiratory tract illness, hypertension, and myocardial infarction) by vignettes relevant to people with mental illness (work, side effects, and medication); and (iv) to achieve conceptual equivalence and logical consistency between the parallel patient and staff versions, staff version wording of items scores in section B and item content was adapted (ISPOR 8). Final versions were then carefully proofread by local CEDAR team members and checked for consistency in order of items and format by the Ulm study team (ISPOR 9). This paper comprises the final report of the entire process of instrument development (ISPOR 10).

### Psychometric evaluation

Following its development, the reliability and validity of the CDMS was examined using data from the study “Clinical Decision Making and Outcome in Routine Care for People with Severe Mental Illness” (CEDAR; ISRCTN75841675). Between November 2009 and December 2010, 588 people with severe mental illness gave informed consent to participate in the CEDAR study which is a naturalistic prospective longitudinal observational study with bimonthly assessments during a 12-month observation period (T0-T6). Participants were recruited from the caseloads of outpatient/community mental health services at six centres throughout Europe: Department of Psychiatry II, Ulm University, Germany (coordinating centre); South London and Maudsley NHS Foundation Trust, London, U.K.; the Department of Psychiatry at Second University of Naples, Italy; the Department of Psychiatry at Debrecen University, Hungary; the Unit for Psychiatric Research at Aalborg Psychiatric Hospital, Denmark; and the Department of General and Social Psychiatry at University of Zurich, Switzerland. Before the start of recruitment, the study protocol was approved by ethics committees in all centres: Ulm University Ethics Commission; Joint South London and Maudsley and Institute of Psychiatry Research Ethics Committee; Ethical Committee of the Second University of Naples, Naples; National Committee on Health Research Ethics, North Denmark Region; Regional and Institutional Ethics Committee, University of Debrecen Medical and Health Science Center; and Kantonale Ethikkommission Zürich. Inclusion criteria were: adult age (18-60 years) at intake, mental disorder of any kind as main diagnosis established by case notes or staff communication using SCID criteria [41,42], presence of severe mental illness (Threshold Assessment Grid [43] ≥ 5 points and illness duration ≥ 2 years); expected contact with mental health services (excluding inpatient services) during the time of study participation; sufficient command of the host country’s language; and capability of giving informed consent. Exclusion criteria were: main diagnosis of learning disability, dementia, substance use or organic brain disorder; cognitive impairment severe enough to make it impossible to give meaningful information on study measures; and treatment by forensic mental health services. Staff were recruited via patients who identified a key professional at baseline. Data were collected using questionnaires (filled in by the patient or his or her key worker) or through interviews conducted by the *CEDAR* research workers. Data entry modes were via computer or paper-pencil forms. See Puschner et al. [16] for further details on rationale and design of the CEDAR study.

#### Measures

The CDMS is a modified version of the “Autonomy Preference Index” [28] adapted for use in mental health care (see above). Patient (CDMS-P) and staff (CDMS-S) versions both have 21 items in three sections: (A) 6 items referring to general preferences regarding patient autonomy in decisions (items #1, #2, #3 and #5 are reversed); (B) 9 items referring to decision making preferences in three scenarios (3 per vignette); and (C) 6 items referring to desire for information (item #19 is reversed). Items in sections A and C are each rated on a five-point Likert scale from “strongly disagree” (0) to “strongly agree” (4). Items in section B are scored from 4 (“Me”) to 0 (“Clinician”) in CDMS-P, and from 4 (“Service user”) to 0 (“Me”) in CDMS-S.

CDMS subscales are *Participation in Decision Making* (PD) which consists of the prorated mean of items in sections A and B (ranging 0-4, with a higher score indicating a higher desire by the service user to be an active participant in decision making), and *Information* (IN) consisting of the prorated mean of items in sections C (ranging 0-4, 0 with a higher score indicating a higher desire by the service user to be provided with information). CDMS total scores were prorated when at least 80% of the items making up a scale had been completed, i.e. at least 12 items of the PD subscale, and at least 4 items of the IN subscale. Categorical sum scores were formulated on the basis of utility where an emphasis was placed on separating categories according to clinical meaningfulness. Categories for PD subscale were Passive (<1.5), Shared (1.5-2.5) and Active (>2.5), and for IN subscale were Low (<2.0), Moderate (2.0-3.0) and High (>3.0). These categories distinguish groups by their ordinal nature but not by a specific value assigned to each category. The CDMS patient and staff versions in all five CEDAR study languages can be downloaded at http://www.cedar-net.eu/instruments.

Two items from the “Clinical Decision Making Involvement and Satisfaction” scale (CDIS, [34]), comprising level of involvement (five point scale from “I made the final decision” through “My clinician and I shared responsibility for making the best decision for me” to “My clinician made the final decision”) and satisfaction (“I am satisfied that I am adequately informed about the issues important to the decision”; five point scale from “strongly disagree” to “strongly agree”).

The Stages of Recovery Inventory (STORI) is a patient-rated 30-item assessment resulting in allocation to one of three stages of recovery (“Moratorium”, “Awareness/Preparation”, and “Rebuilding/Growth”) [44,45].

### Analysis

Distribution characteristics were examined by means of item analysis (means, standard deviation, skewness, kurtosis, missing values) and Q-Q plots. The Q-Q plot is a graphical method for comparing two distributions. Subscale scores were plotted against a theoretical normal distribution (also called normal probability plot) where points should approximately form a straight line.

Reliability was examined by calculating internal consistency (Cronbach’s alpha) including confidence intervals to increase precision of estimates [46], and by analysis of discriminability. Interpretation of Cronbach’s alphas followed the recommendations of Nunnally [47] (“reliabilities of 0.7 or higher will suffice”, p. 245) and George and Mallery [48] (acceptable: > .7; good: > .8; excellent: > .9). Discriminatory power was investigated by calculating the Corrected Item-Total Correlation (CITC) which gives the correlation between a given item and the sum score of the other items making up the scale. CITCs above .30 are considered adequate [49].

Furthermore, the continuous as well as the utility (categorised) CDMS total scores were analysed via Spearman correlations to establish relations among CDMS subscales, convergent validity (with two CDIS items), concurrent validity (with STORI recovery stage) and stability over one year.

## Results

### Sample

A total of 708 patients were screened for eligibility, of whom 588 were included. Reasons for exclusions were not meeting inclusion criteria (n=120), refusal to participate (n=78), and other reasons (n=3: one suicide, one deceased, one too anxious to participate). Patient participants are described in Table 1. Mean GAF score for participants indicates serious symptomatology and social disability, indicating that the TAG threshold had successfully resulted in a sample of participants who can be characterised as having severe mental illness.

**Table 1:** Sociodemographic and clinical characteristics of patient participants (N=588)

Study centre	
Ulm, *n (%)*	112 (19.05)
London, *n (%)*	85 (14.46)
Naples, *n (%)*	101 (17.18)
Debrecen, *n (%)*	97 (16.49)
Aalborg, *n (%)*	98 (16.67)
Zurich, *n (%)*	95 (16.16)
Gender; female, *n (%)*	307 (52.21)
Age; years, M (SD)	41.69 (10.74)
Married; *n (%)*	149 (25.38)
Ethnic group; Caucasian; *n (%)*	552 (94.04)
Years in school; M (SD)	10.43 (1.88)
Living alone; *n (%)*	231 (39.55)
Paid or self employed; *n (%)*	110 (18.74)
Receiving state benefits; *n (%)*	425 (72.40)
Illness duration; years, *M (SD)*	12.51 (9.27)
Diagnosis	
Psychotic disorder, *n (%)*	269 (45.75)
Mood disorder, *n (%)*	200 (34.01)
Other, *n (%)*	119 (20.24)
TAG; *M (SD)*	7.54 (2.24)
GAF; *M (SD)*	49.03 (10.96)
STORI-30	
(1) Moratorium, *n (%)*	115 (19.79)
(2) Awareness and Preparation, *n (%)*	145 (24.96)
(3) Rebuilding and Growth, *n (%)*	321 (55.25)

*Notes:* Missing values: N=1 (married, ethnic group, work, benefits), N=4 (living), N=7 (STORI), N = 11 (school), N=29 (GAF).

Participating staff were in their mid-40s on average, and mean time of working in mental health services was 15 years. The “other” category for professions included nurse, district nurse, support time and recovery worker, and psychiatric trainee (see Table 2).

**Table 2:** Sociodemographic and professional characteristics of staff participants (N=213)

Study centre	
Ulm, *n (%)*	48 (22.54)
London, *n (%)*	38 (17.84)
Naples, *n (%)*	17 (7.98)
Debrecen, *n (%)*	8 (3.79)
Aalborg, *n (%)*	59 (27.69)
Zurich, *n (%)*	43 (20.19)
Gender; female, *n (%)*	128 (61.84)
Age; years, *M (SD)*	46.03 (10.47)
Profession	
Psychiatrist, *n (%)*	75 (36.41)
Psychologist, *n (%)*	19 (9.22)
Social Worker, *n (%)*	11 (5.34)
Other, *n (%)*	101 (49.03)
Working in outpatient mental health services; years, *M (SD)*	9.41 (8.44)
Working in mental health services; years, *M (SD)*	14.99 (9.66)
Number of patients in study; *M (SD)*	2.76 (4.46)

*Notes.* Missing values: N = 6 (gender), N=7 (profession), N = 54 (working outpatient), N = 41 (working mental health).

### Item characteristics and reliability

#### Patient version

As shown in Table 3 (left section), range of all items of the CDMS-P PD was quite homogenous (*SD* = 0.89 – 1.36). Items with the most extreme difficulties were #4 and #5, indicating that participants used 32.7% (2.61 - 0.97/5) of the whole range of the 5-point scale. Skewness of items averaged at 0.14 (*SD* = 0.37; *range* = -0.51 [#4] – 1.05 [#5]), and kurtosis at -0.48 (*SD* = 0.64; *range* = -1.29 [#1] – 0.81 [#7]), respectively. Number of missing values ranged from 0.17 – 1.87%. Mean discriminative power (corrected item-total correlation) was 0.48 (SD = 0.13), with item #4 falling below the cut-off of 0.3. Cronbach’s α ranged between .87 and .89 regardless of the omission of any item.

**Table 3:** Item characteristics CDMS Patient (N = 588) and Staff (N = 570) versions

	***Patient version***			***Staff version***		
***Participation in Decision Making (PD) sub-scale***	***Mean (SD)***	***CITC***	***α***	***Mean (SD)***	***CITC***	***α***
1 Important decisions.^a^	1.95 (1.36)	0.524	0.837	2.71 (1.27)	0.767	0.870
2 Comply with clinician’s advice.^a^	1.58 (1.18)	0.520	0.837	2.20 (1.19)	0.668	0.876
3 Treatment in the clinic.^a^	1.85 (1.34)	0.552	0.835	2.58 (1.22)	0.695	0.874
4 Every day problems.	2.61 (1.08)	0.140	0.857	2.38 (1.10)	0.302	0.894
5 More control when worsening.^a^	0.97 (0.98)	0.445	0.842	1.13 (0.94)	0.453	0.885
6 See clinician how often.	2.00 (1.23)	0.413	0.844	1.39 (0.96)	0.341	0.890
7 Return to work.	2.24 (0.89)	0.453	0.842	1.95 (0.60)	0.545	0.882
8 Suitable occupation.	2.46 (0.91)	0.368	0.845	1.92 (0.55)	0.325	0.888
9 Amount of work.	2.42 (0.97)	0.405	0.844	2.04 (0.63)	0.508	0.883
10 See a doctor.	2.40 (1.29)	0.393	0.845	2.17 (0.95)	0.485	0.884
11Dosage of medication.	1.35 (1.06)	0.573	0.835	2.66 (0.73)	0.697	0.876
12 Another medication.	1.28 (1.02)	0.612	0.833	2.68 (0.77)	0.701	0.876
13 Medication at all.	1.53 (1.20)	0.617	0.831	2.60 (0.76)	0.741	0.874
14 Form of medication.	1.83 (1.26)	0.555	0.835	2.18 (0.78)	0.563	0.881
15 Duration of medication.	1.24 (1.04)	0.663	0.830	2.70 (0.78)	0.737	0.874
***Information (IN) sub-scale***	***Mean (SD)***	***CITC***	***α***	***Mean (SD)***	***CITC***	***α***
16 Informed about the facts.	3.14 (0.94)	0.506	0.660	2.63 (0.89)	0.350	0.717
17 Know exactly.	3.29 (0.86)	0.617	0.630	3.04 (0.74)	0.638	0.631
18 Explain purpose.	3.41 (0.74)	0.623	0.639	3.43 (0.63)	0.550	0.665
19 Information when asked for.^a^	2.64 (1.23)	0.121	0.807	3.03 (0.91)	0.232	0.753
20 Side effects.	3.23 (0.97)	0.467	0.672	2.55 (0.97)	0.516	0.664
21 Various treatment methods.	3.39 (0.80)	0.587	0.643	3.25 (0.70)	0.565	0.656

*Notes:*^a^ 5 items reverse coded for analysis; *CITC*: Corrected Item-Total Correlation; α = α after item deletion; Ns for CITC and α: 573 for PD-P, 583 for IN-P, 555 for PD-S, and 568 for IN-S.

For the CDMS-P IN, participants used 15.5% of the scale range. Skewness was negative for all items (M = -1.35; SD = 0.29; *range* = -0.82 [#19] – -1.56 [#18]; kurtosis: M = 2.00; SD = 1.47; *range* = -0.35 [#19] – 3.50 [#18]), and missing values ranged from 0.17 – 0.34%. Cronbach’s α substantially increased when deleting item #19 which also showed low CTIC, so this item is deleted from the sub-scale in subsequent analysis.

#### Staff version

As also shown in Table 3 (right section), range of all items of the CDMS-S PD was also quite homogenous (*SD* = 0.55 – 1.27). Items with the most extreme difficulties were #1 and #5, indicating that participants used 31.6% of the 5-point range. Mean skewness of items was -0.10 (SD = 0.50; *range* = -0.81 [#1] – 1.13 [#5]), and mean kurtosis was 0.42 (SD = 1.45; *range* = -0.92 [#2] – 3.38 [#7]). Number of missing values ranged from 0 – 1.58%, and mean discriminative power was 0.57 (SD = 0.16), with some items (#4, #8) barely meeting the cut-off of adequate discriminability. Consistent with the patient version, Cronbach’s α ranged between .87 and .89 regardless of the omission of any item.

For the CDMS-S IN, participants used 17.5% of the scale range, and skewness was negative for all items (M = -0.74; SD = 0.33; *range* = -1.09 [#19] – -0.23 [#20]; kurtosis: M = 0.86; SD = 1.04; *range* = -0.70 [#20] – 1.89 [#18]), and missing values ranged from 0 – 0.35%. Furthermore, as in the patient version, Cronbach’s α was highest when deleting item #19 which also showed poor CITC, so this item is deleted from the sub-scale in subsequent analysis.

**Figure 1:**
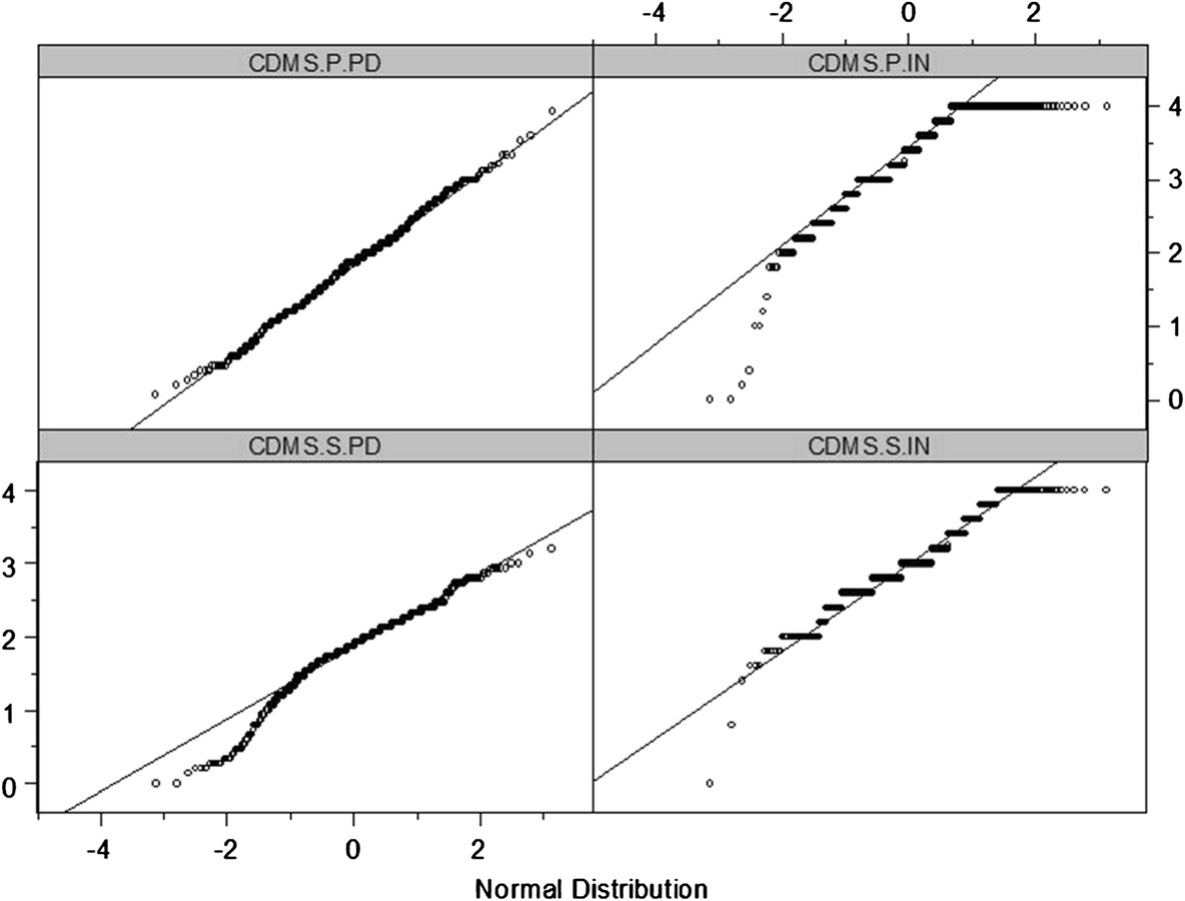
Q-Q Plots of patient (above) and staff ratings (below) of CDMS subscales Participation in Decision Making (left) and Information (right).

Descriptives of all four CDMS subscale scores without item #19 in both patient and staff versions as well as Cronbach’s α’s including their confidence intervals are shown in the upper part of Table 4. As can be seen in Figure 1,0 for both patient and staff versions, PD scores are approximately normally distributed which is not the case for IN scores which are distinctly skewed to the left.

**Table 4:** CDMS subscale scores at baseline and follow-up

	***Scale***	***Mean (SD)***	***N***	***α (CI 95%)***	***Utility:***	***passive, N (%)***	***shared, N (%)***	***active, N (%)***
**Baseline**	*PD Patient*	1.84 (0.64)	586	0.849 (0.830 - 0.866)		175 (29.86)	319 (54.44)	92 (15.69)
	*PD Staff*	1.83 (0.57)	563	0.888 (0.874 - 0.901)		124 (22.02)	397 (70.51)	42 (7.46)
					***Utility:***	***low, N (%)***	***moderate, N (%)***	***high, N (%)***
	*IN Patient*	3.29 (0.65)	587	0.808 (0.782 - 0.831)		21 (3.58)	207 (35.26)	359 (61.16)
	*IN Staff*	2.98 (0.56)	570	0.753 (0.720 - 0.784)		45 (7.89)	324 (56.84)	201 (35.26)
	*Scale*	*Mean (SD)*	*N*	*ρ*_*tt*_	***Utility:***	***passive, N (%)***	***shared, N (%)***	***active, N (%)***
**1 year**	*PD Patient*	1.87 (0.68)	514	*ρ=*0.69; *p<*0.001; *N*=512		152 (29.57)	276 (53.69)	86 (16.73)
				Utility*: *ρ=*0.64; *p<*0.001				
	*PD Staff*	1.84 (0.57)	491	*ρ=*0.79; *p<*0.001; *N*=485		114 (23.22)	337 (68.64)	40 (8.15)
				Utility*: *ρ=*0.66; *p<*0.001				
					***Utility:***	***low, N (%)***	***moderate, N (%)***	***high, N (%)***
	*IN Patient*	3.31 (0.55)	515	*ρ=*0.36; p<.001; *N*=514		12 (2.33)	213 (41.36)	290 (56.31)
				Utility*: *ρ=*0.24; *p*<0.001				
	*IN Staff*	3.05 (0.57)	495	*ρ=*0.44; p<0.001; *N*=490		27 (5.45)	270 (54.55)	198 (40.00)
				Utility*: *ρ=*0.31; p<.001				

*Notes: CI* = confidence interval; Utility: Clinical utility categorical data; * = Ns as previous line.

As also shown in Table 4, clinical utility categorical scores show that the vast majority of both patients and staff members prefer shared (rather than passive or active) participation in decision making, while need for information was mostly high in patients and predominantly moderate in staff.

### Stability

Descriptives for CDMS subscales at one-year follow-up are also shown in Table 4. Distribution of clinical utility categorical scores was similar to baseline data. Paired t-tests showed that, compared to T0, scores one year later were higher for IN staff (t_df=489_ = -2.59, p = .010), and not different for the other scales (PD patient: t_df=511_ = -1.93, p = .054; IN patient: t_df=513_ = -0.21, p = .830; PD staff: t_df=489_ = 0.26, p = .798). Spearman correlation coefficients between baseline and one-year follow-up of the four scales ranged between 0.36 and 0.79 (and 0.24 and 0.66 for the clinical utility categorical scores), and were higher for the PD scales in than for the IN scales in both patient and staff versions.

### Validity

Spearman correlations between PD and IN subscales were close to 0 for both patients and staff versions (see Table 5).

**Table 5:** Relations among CDMS subscales and convergent validity (Spearman)

***Scale***	***IN***	***CDIS involvement***	***CDIS information***	***STORI***
***Continuous***				
*PD Patient*	*ρ=*0.01	*ρ=*-0.27		*ρ=*0.04
	*p*=0.837	*p*<0.001		*p*=0.319
	*N*=586	*N*=445		*N*=580
*PD Staff*	*ρ=*0.14	*ρ=*-0.43		
	*p*=0.001	*p*<0.001		
	*N*=563	*N*=417		
*IN Patient*			*ρ=*0.31	*ρ=*0.12
			*p*<.001	*p*=.003
			*N*=446	*N*=580
*IN Staff*			*ρ=*0.27	
			*p*<0.001	
				*N*=422	
Utility*					
*PD Patient*	*ρ=*0.06	*ρ=*-0.26		*ρ=*0.07	
	*p*=0.181	*p*<0.001		*p*=0.093	
*PD Staff*	*ρ=*0.08	*ρ=*-0.39			
	*p*=0.051	*p*<0.001			
*IN Patient*			*ρ=*0.26	*ρ=*0.11	
			*p*<0.001	*p*=0.008	
*IN Staff*			*ρ=*0.18		
			*p*<0.001		

*Notes:* *Ns as above.

As also shown in Table 5, correlations were moderate for PD with the CDIS involvement item in both patients and staff. Correlation of IN with the CDIS information item was also moderate in patients, but the two variables were hardly related in the staff version. Finally, analysis of concurrent validity showed that CDMS-P PD was unrelated to recovery, while CDMS-P IN scores increased with a higher STORI stage (F_df = 2;577_ = 3.54; p = .030; also see Table 4). Correlations using the clinical utility categorical scores yielded similar results.

## Discussion

This paper reports on instrument development and psychometric properties of the Clinical Decision Making Style Scale (CDMS) which consists of patient (CDMS-P) and staff (CDMS-S) versions which are structured identically with wording changed to reflect the different perspectives.

Instrument development followed current state-of-the-art recommendations to ensure consistently high standards across study sites in preparing the source version and the final versions in five languages. Throughout this process, special efforts were made to use a participatory approach, i.e. expert advice from patients and staff was systematically sought from the start, in order to generate an instrument which is feasible and meaningful to its users. High face validity and completion rates of no less than 98% on any CDMS item indicate that this process has been successful.

Item analysis showed that items of the PD subscale were approximately normally distributed and that participants used a large portion of the 5-point scale. In contrast, the items in the IN subscale were distinctly left skewed and consequently also participants’ use of the scale range was rather restricted. Item-level findings were consistent with the total scores, which for the PD subscale in both patients and staff showed approximate normal distribution, while both IN subscale totals were distinctly left skewed. This pattern is in line with Giersdorf and colleagues [50] who also reported that IN items showed little variance and a ceiling effect in 646 people with different chronic conditions. In line with Ende and colleagues [28], this indicates that patients have a strong interest in being well informed. Additionally it shows that mental health professionals acknowledge a high need for information among service users.

Furthermore, categorising the subscale totals according to clinical utility criteria showed that both patients and staff members preferred shared (rather than passive or active) participation in decision making, while need for information was high from the patient perspective and moderate from the staff perspective.

### Reliability

Reliability indices were satisfactory to excellent for all items in the PD subscale. This was also the case in both patient and staff versions of the IN subscale, apart from one item (#19) which showed low discriminatory power and contributed negatively to internal consistency in both patient and staff versions of the IN subscale. After deletion of this item, internal consistency (Cronbach’s α’s) including the lower bounds of the 95% confidence intervals were at least adequate, and mainly good. These findings are in line with previous research on the API which has also identified some items with poor reliability [33] and reported similarly good internal consistency [28,32] in people with various conditions. However, in a study with people with schizophrenia [29], the API’s internal consistency was poor(α = 0.57), justifying the efforts made during the CDMS development process to modify and maximise its meaningfulness for people with severe mental illness.

Stability over one year was high for PD in both patients and staff, and moderate for IN. Overall, this finding shows that, as intended, the CDMS measures a relatively constant trait-like component of clinical decision making.

### Validity

PD and IN subscales did not correlate. This indicates convergent validity and shows that, as in the original API, preferences for participation in decision making are independent of preferences for information. This study adds that this is also the case in people with severe mental illness and in mental health professionals. Furthermore, convergent validity could be established for the PD subscale for both patient and staff versions, and for the IN subscale for the patient version. However, correlations with the corresponding CDIS items were only moderate, and the IN subscale did not correlate with the corresponding CDIS information item. When interpreting these finding on convergent validity, it should be borne in mind that CDMS taps into general aspects of decision making style, while CDIS rates involvement and information relating to a specific decision making encounter. Finally, concurrent validity was demonstrated for the CDMS-P IN by showing that the patient-rated need for information increased with a higher stage of recovery, while the CDMS-P PD subscale was not related to recovery. Thus, concurrent validity could only partially be demonstrated. This finding raises the question about the relationship between recovery and participation in decision making.

Analysis on all indices of reliability and validity for the categorised clinical utility categories yielded results similar to the analysis of the continuous CDMS variables. This finding indicates the adequacy of the chosen cut-off points.

### Limitations

This study has several limitations. First, test-retest reliability in the strict sense, with participants filling in the scale again shortly after initial completion has not been examined. Test-retest reliability should be tested for shorter intervals. Second, evaluation of validity was made difficult because clinical decision-making style is a specific concept, making identification of comparator scales problematic. Third, there are weaknesses in sample generalizability. In the instrument development, convenience samples were chosen as participants of the focus group. Thus, the samples may not truly reflect the mentally ill population. The same issue arises for staff participants in focus groups. Fourth, a pilot phase between instrument development and administration of the instruments in the CEDAR study would have been worthwhile. Finally, future studies might consider sampling other populations containing native speakers of the five languages.

## Conclusion

This study investigated the psychometric properties of the Clinical Decision Making Style (CDMS) scale which measures general and specific preferences for decision making (subscale *Participation in Decision Making* - PD) as well as preferences regarding the provision of information to the patient (subscale *Information* - IN) from the perspectives of people with severe mental illness (CDMS –P) and mental health professionals (CDMS –S). The subscales reliably measured distinct characteristics of decision making, which showed relative stability over time. Validity was demonstrated to some extent and needs further investigation. Overall, the psychometric properties of the CDMS are satisfactory making it possible to further examine the relation between clinical decision making and outcome in the treatment of people with severe mental illness across Europe.

## Abbreviations

CDMS: Clinical Decision Making Style Scale; CDMS-P: Clinical Decision Making Style Scale Patient Version; CDMS-P: Clinical Decision Making Style Scale Staff Version; PD: Participation in Decision Making (CDMS subscale); IN: Information (CDMS subscale); API: Autonomy Preference Index.

## Competing interests

The authors declare that they have no competing interests.

## Authors' contributions

PN, SL and BP coordinated instrument development across sites. PN, HJ, DG, TI, MKB, HOS, and AB collected data for instrument development and for the main study. PN, SL and KA analysed the qualitative data from instrument development. BP drafted the manuscript and carried out the quantitative data analysis to establish psychometric properties. PN, MS, AF, AE, and WK revised the manuscript critically for important intellectual content. All authors have given final approval of the version to be published.

## Pre-publication history

The pre-publication history for this paper can be accessed here:

http://www.biomedcentral.com/1471-244X/13/48/prepub
